# A novel surgical technique for prevention of Ahmed glaucoma valve tube exposure: long scleral flap augmented with Tenon advancement and duplication

**DOI:** 10.1186/s12886-018-0907-y

**Published:** 2018-08-31

**Authors:** O. M. Gedar Totuk, K. Kabadayi, A. Colakoglu, N. Ekizoglu, U. Aykan

**Affiliations:** 1 Department of Ophthalmology, Bahcesehir University Faculty of Medicine, Yenisahra Mah. Batman Sok. No: 66-68 Sahrayicedit, Kadikoy, Istanbul, Turkey; 2 Bahcesehir University Faculty of Medicine, Istanbul, Turkey; 3 FEBO, Dunyagoz Hospital, Istanbul, Turkey

**Keywords:** Refractory glaucoma, Glaucoma valve tube exposure, Glaucoma valve implantation, Long scleral flap, Tenon duplication, Ahmed glaucoma valve

## Abstract

**Background:**

To describe a new technique and present its long-term outcome for prevention of Ahmed glaucoma valve (AGV) tube exposure in patients with refractory glaucoma.

**Methods:**

Twenty-seven eyes of 24 patients (mean age, 50 years; age range, 16–78 years; 8 females, 16 males) with refractory glaucoma who had the AGV implant were retrospectively reviewed. For AGV implantation, a long scleral flap combined with Tenon advancement and duplication was used. In this technique, a long scleral flap is created to completely cover the extraocular part of valve’s tube, and the flap surface is covered with duplicated Tenon’s tissue. The average follow-up after AGV implantation was 21.7 months (range, 12–36 months).

**Results:**

The mean intraocular pressure before the operation, which was 44.1 mmHg (range, 26–62 mmHg), decreased to 14.2 mmHg (range, 8–20 mmHg) at the last follow-up visit, showing 67% reduction with AGV implantation. The mean number of antiglaucomatous medications was 4.1 before the AGV implantation and decreased to 0.9 after the operation, showing 88% reduction. In 14 eyes (51.9%), there was no change in the best corrected visual acuity (BCVA), and in 11 eyes (40.7%), the BCVA increased by 2 lines on the Snellen chart postoperatively. No patient developed postoperative hypotony, flat anterior chamber, diplopia, strabismus, erosion or exposure of the tube, or tube/plate migration.

**Conclusions:**

The long scleral flap augmented with Tenon advancement and duplication is an effective and safe surgical technique for the implantation of AGV and preventing tube exposure in cases of refractory glaucoma.

## Background

The management of refractory glaucoma is often challenging. The common approach is to reduce intraocular pressure by using glaucoma drainage devices. In comparison to glaucoma surgeries, the Ahmed glaucoma valve (AGV) implantation has been found to be more effective and safe method for reducing intraocular pressure in patients with refractory glaucoma [1–4]. Implantation of AGV can be used as a primary treatment option in refractory glaucoma or after failed conventional filtration surgeries [1].

Early and late hypotony, shallow anterior chamber, corneal-lenticular touch, choroidal detachment, hypotony maculopathy, anterior chamber hyphema, suprachoroidal hemorrhage, peripheral iris synechiae, capsule fibrosis around plate, erosion and exposure of the tube or plate, extrusion of the implant, endophthalmitis, and cataract are some of the complications associated with AGV [2, 5]. AGV tube erosion almost always occurs at the proximal limbal conjunctiva if left uncovered, subsequently leading to exposure of the tube and increased risk of endophthalmitis [5, 6]. Even when the tube is covered with graft materials like pericardial patch grafts, donor fascia lata graft, cadaveric dura mater, and preserved human sclera, the risk of exposure still exists. Grafts also carry the risk of virus transmission, corneal dellen formation, scleral melting, and high financial burden [7, 8]. Thus, there is no perfect surgical technique to prevent AGV tube exposure [9].

We propose a new technique that completely buries the tube within the sclera using a covering of a long scleral flap with Tenon advancement and duplication to prevent AGV tube exposure in patients with refractory glaucoma. In this study, we aimed to present this technique and its long-term outcomes with regard to prevention of AGV tube exposure in patients with refractory glaucoma.

## Methods

### Study patients

Twenty-seven eyes of 24 patients (mean age, 50 years; age range, 16–78 years; 8 females, 16 males) with refractory glaucoma who underwent AGV implantation in our clinic between March 2014 and January 2018 were retrospectively reviewed. This study describes a novel surgical technique, however, this technique has been applied in our clinic within the routine clinical practice since 2014. Therefore, this study was performed as a retrospective review of patient records. For AVG implantation, a long scleral flap combined with Tenon advancement and duplication technique was used in all patients. All patients underwent a comprehensive assessment and ophthalmologic examination, including measurement of best corrected visual acuity (BCVA) on the Snellen chart and intraocular pressure (IOP) with applanation tonometry. Of the 27 eyes, 5 had neovascular glaucoma with proliferative diabetic retinopathy, 5 had primary open angle glaucoma, 2 had congenital glaucoma, 15 had secondary glaucoma. Nine eyes (33.3%) had phakia, 1 (3.7%) had aphakia, and 17 (63%) had pseudophakia. The demographics, diagnosis, and ophthalmological findings of each eye before AGV implantation are summarized in Table 1.

**Table 1:** Patient demographics and clinical data before AGV implantation surgery

Patient	Age	Sex	Side	Diagnosis	Lens	Additional treatment
1	> 50	F	R	NVG + PDR	Phakic	AC Lucentis
2	> 50	F	L	NVG + PDR	Phakic	AC Lucentis
3	> 50	M	R	NVG + PDR	Pseudophakic	AC Lucentis
4	11–20	M	L	SEC GL (PPV + silicon) + PVR	Pseudophakic	AC Avastin
5	41–50	M	R	SEC GL (PPV + silicon) + PDR	Pseudophakic	
6	> 50	F	R	NVG + PDR	Pseudophakic	AC Avastin
7	> 50	F	L	NVG + PDR	Pseudophakic/ Subluxated	AC Avastin
8	> 50	M	R	PAOG/failed SLT	Pseudophakic/ Degen myopia	–
9	> 50	M	R	PAOG	Phakic	–
10	> 50	M	L	SEC GL (PPV + silicon) + PDR	Pseudophakic	–
11	11–20	F	L	SEC GL (CONG CAT/SEC IOL implant/opaque cornea)	Pseudophakic	–
12	21–30	M	R	SEC GL (PPV + silicon)	Phakic	–
13	31–40	F	L	CONG GL	Phakic/Bullous keratopathy	–
14	41–50	M	R	SEC GL (PPV + silicon)	Pseudophakic	–
15	> 50	F	R	SEC GL (Keratoplasty)	Pseudophakic	–
16	21–30	M	L	SEC GL (PPV + Silicon)	Pseudophakic	–
17	41–50	F	L	Traumatic GL/Failed hydrus	Phakic/Bullous keratopathy	–
18	> 50	F	R	Uveitic GL (HSV)	Phakic	–
19	> 50	M	L	SEC GL (PPV + Silicon) + PDR	Phakic	AC Avastin
20	> 50	M	L	SEC GL (anterior chamber IOL + bullous keratopathy)	Pseudophakic	–
21	41–50	M	L	SEC GL (PPV + silicon) + PDR	Pseudophakic	AC Avastin
22	> 50	M	L	SEC GL (PPV + silicon)	Pseudophakic	–
23	31–40	M	L	CONG GL	Pseudophakic	–
24	41–50	M	R	POAG/RP	Pseudophakic	–
25	41–50	M	L	POAG/RP	Pseudophakic	–
26	> 50	M	R	POAG/failed TRAB	Phakic	–
27	21–30	F	L	Traumatic+aphakic GL	Aphakic	–

*AC* anterior chamber, *CAT* cataract, *CONG* congenital, *F* female, *GL* glaucoma, *HSV* herpes simplex virus, *IOL* intraocular lens, *L* left, *M* male, *NVG* neovascular glaucoma, *PDR* proliferative diabetic retinopathy, *POAG* primary open angle glaucoma, *PPV* pars plana vitrectomy, *PVR* proliferative vitreoretinopathy, *R* right, *RP* retinitis pigmentosa, *SEC* secondary, *SLT* selective laser trabeculoplasty, *TRAB* trabeculectomy

This study was approved by the Institutional Ethics Committee of Bahcesehir University (Dec/6th/2017; 2017–19/04) and conducted in accordance with the latest version of the Declaration of Helsinki. According to the Regulation on Clinical Studies of Drugs and Biological Products in Turkey (no: 29474), which was updated on 13.09.2015, retrospective studies are not subject to the requirement of informed consent of patients. The Institutional Ethics Committee of Bahcesehir University, which operates in accordance with this regulation, waived the requirement of informed consent for this study (Dec/6th/2017; 2017–19/04).

### Surgical technique

**Fig. 1:**
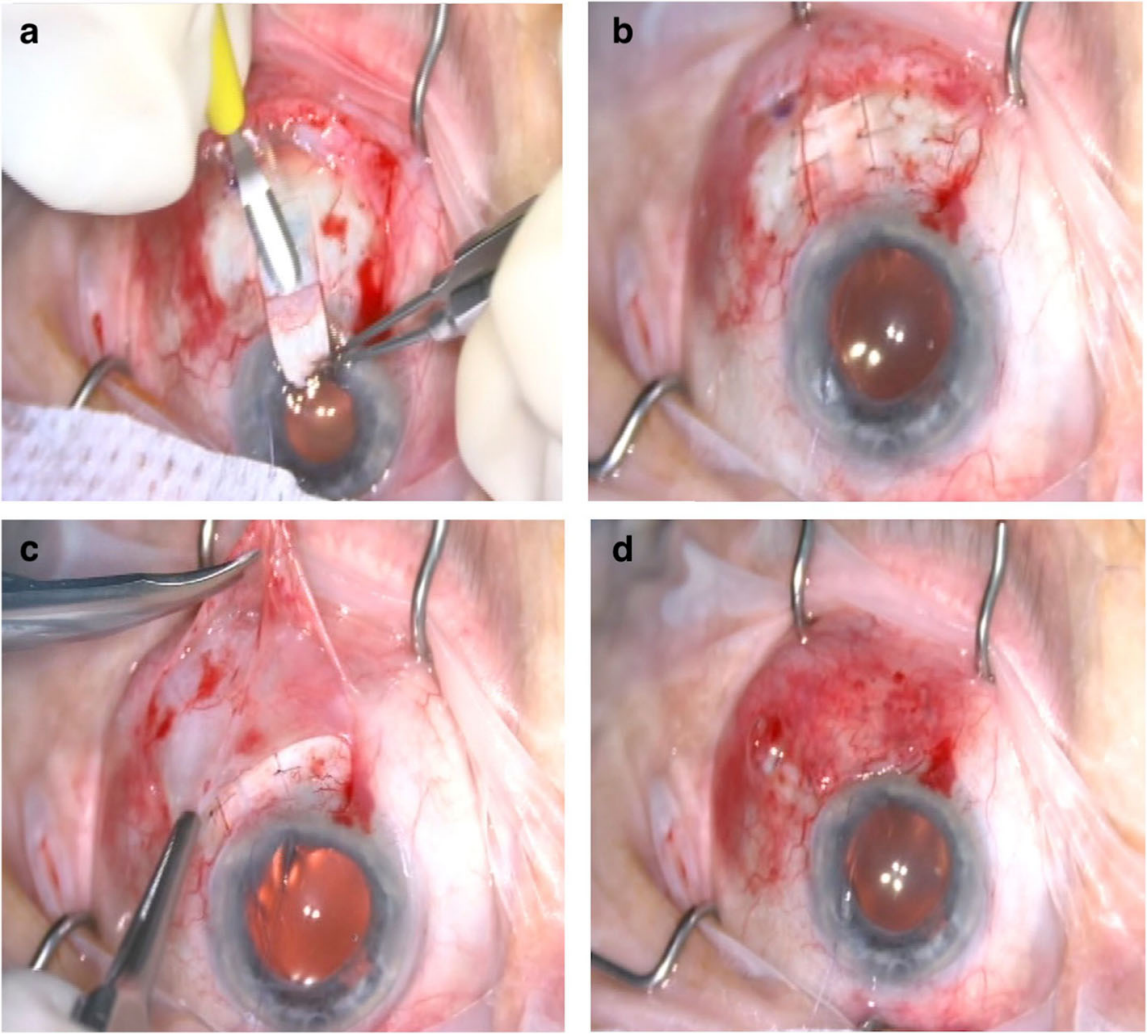
The long scleral flap combined with tenon advancement and duplication technique. First, a 10 mm long scleral flap is prepared with bevel up crescent knife between the two scleral incisions (**a**). The AC is entered with a 23G needle, tube was inserted into the AC, then the scleral flap was sutured with with 10/0 monofilament nylon sutures (**b**). Tenon advancement and duplication technique over the long scleral flap that covered the tube is applied (**c**). Finally, the conjunctiva is anchored to the limbus with 8/0 interrupted vicryl sutures (**d**)

A fornix-based conjunctival flap was prepared with one perpendicular relaxing incision in the superotemporal quadrant for the right eye or superonasal quadrant for the left eye because the surgeon is right handed. The AGV model FP7 (New World Medical Inc., Rancho Cucamonga, CA, USA) was used for all surgeries. The implant was examined for integrity and primed by injecting 1 cc of balanced salt solution through the drainage tube. The AGV implant was inserted under the conjunctiva and Tenon’s capsule and sutured to the sclera at a distance of about 10 mm from the limbus with a 6/0 Vicryl suture. Two approximately 10-mm-long parallel scleral incisions were made starting from the limbus ending at the plate, of half thickness (around 250-μm thick), throughout the tube tract. Then, a 10-mm-long scleral flap was prepared with a bevel up crescent knife (Fig. 1a). The drainage tube was trimmed to the appropriate length permitting a 2–3-mm insertion in the anterior chamber (AC) with the beveled-up edge. The AC was then entered 2 mm posteriorly to the limbus, under the scleral flap with a 23-G needle. The needle tract was parallel to the plane of the iris. The bevel-up trimmed tube was inserted into the AC through the needle tract without making contact with the iris or corneal endothelium. Then, the scleral flap was sutured with 10/0 monofilament nylon sutures (Fig. 1b). Tenon advancement and duplication by blunt dissection into two layers were applied with the long scleral flap that was used to cover the tube (Fig. 1c). Finally, the conjunctiva was anchored to the limbus with 8/0 interrupted Vicryl sutures (Fig. 1d). A subconjunctival injection of corticosteroids and antibiotics was administered at the end of the procedure. Topical corticosteroids, nonsteroidal anti-inflammatory drugs, cycloplegics, and antibiotic regimen was started for all patients postoperatively.

AGV implantation with a long scleral flap covering with Tenon advancement and duplication surgical technique was combined with intracameral anti-VEGF injections in 8 patients with proliferative diabetic retinopathy (Table 1). A single surgeon (U.A.) performed all the operations.

### Statistical analysis

The study data were summarized for each eye using descriptive statistics such as mean, range, standard deviation, frequency, and percentage.

## Results

The mean follow-up period after AGV implantation was 21.7 months (range, 12–36 months) (Table 1). In 2 eyes (7.4%), the BCVA decreased postoperatively by 2 lines on the Snellen chart owing to suprachoroidal hemorrhage and uveitic reactivation. In 14 eyes (51.9%), there were no changes in the BCVA, and in 11 eyes (40.7%), the BCVA increased by 2 lines postoperatively (Table 2).

**Table 2:** Patient clinical data after AGV implantation surgery

Patient	Follow-up (Months)	Preop BCVA	Postop BCVA	Preop IOP	Postop highest IOP	Postop IOP	IOP % Reduction	Number of preop drugs	Number of postop drugs	Complications	SEC operation
1	36	1MCF	0.3	50	34	16	0.68	4	0	Tube occlusion by iris, SEC CAT	IV Lucentis, PHACO, tube repositioning
2	36	1MCF	0.1	34	26	18	0.47	4	3	Hyphema	IV Lucentis, PHACO, AC Lavage
3	16	1MCF	0.2	34	22	12	0.65	5	2	None	0
4	13	HM	HM	34	17	17	0.5	3	0	None	0
5	12	*p*+	HM	54	17	10	0.81	4	0	None	0
6	27	1MCF	0.3	52	22	17	0.67	5	3	IVH	IV Lucentis, PPV
7	27	HM	0.05	54	18	10	0.81	5	0	IVH, hyphema	AC Lavage, IV Lucentis, PPV
8	20	*p*+	*p*+	40	30	19	0.53	4	0	Tenon’s cyst	Cyst drainage, cystectomy
9	12	0.4	0.6	31	10	8	0.74	3	0	None	0
10	12	NLP	NLP	57	24	17	0.7	5	3	None	0
11	24	NLP	NLP	50	30	16	0.68	5	0	None	Diode CPC
12	23	HM	0.05	38	28	10	0.74	3	0	SEC CAT	Silicon extraction, PPV
13	24	NLP	P-	32	28	17	0.47	3	2	Tenon’s cyst	Cyst drainage, cystectomy
14	27	5MCF	5MCF	45	24	15	0.67	6	4	None	0
15	36	0.16	0.6	48	26	16	0.67	6	2	None	0
16	15	NLP	NLP	51	25	12	0.76	4	0	Choroidal detachment	0
17	12	NLP	NLP	51	22	18	0.65	3	2	None	0
18	18	1MCF	1MCF	62	35	20	0.68	4	1	HSV uveitic activation and tube occlusion	0
19	24	2MCF	2MCF	50	10	10	0.8	2	0	None	0
20	27	NLP	NLP	38	28	15	0.61	4	2	None	0
21	24	NLP	NLP	55	12	10	0.82	4	0	None	0
22	18	0.2	0.05	57	28	20	0.65	5	0	Tube occlusion with silicon oil	0
23	18	0.05	HM	38	24	9	0.76	6	0	Suprachoroidal hemorrage, coroidal detachment	PPV
24	22	0.7	1	26	17	13	0.5	4	0	None	0
25	22	0.5	0.7	38	16	11	0.71	4	0	None	0
26	14	0.05	0.1	32	18	9	0.72	4	0	None	0
27	27	HM	HM	40	20	18	0.55	2	0	None	0

*AC* anterior chamber, *BCVA* best corrected visual acuity, *CAT* cataract, *CPC* cyclophotocoagulation, *HM* hand motions, *HSV* herpes simplex virus, *IOP* intraocular pressure, *IV* intravitreal, *IVH* intravireal hemorrhage, *MCF* meters counting fingers, *NLP* no light perception, *P* perception, *PHACO* phacoemulsification, *Postop* postoperative, *PPV* pars plana vitrectomy, *Preop* preoperative, *SEC* secondary

**Fig. 2:**
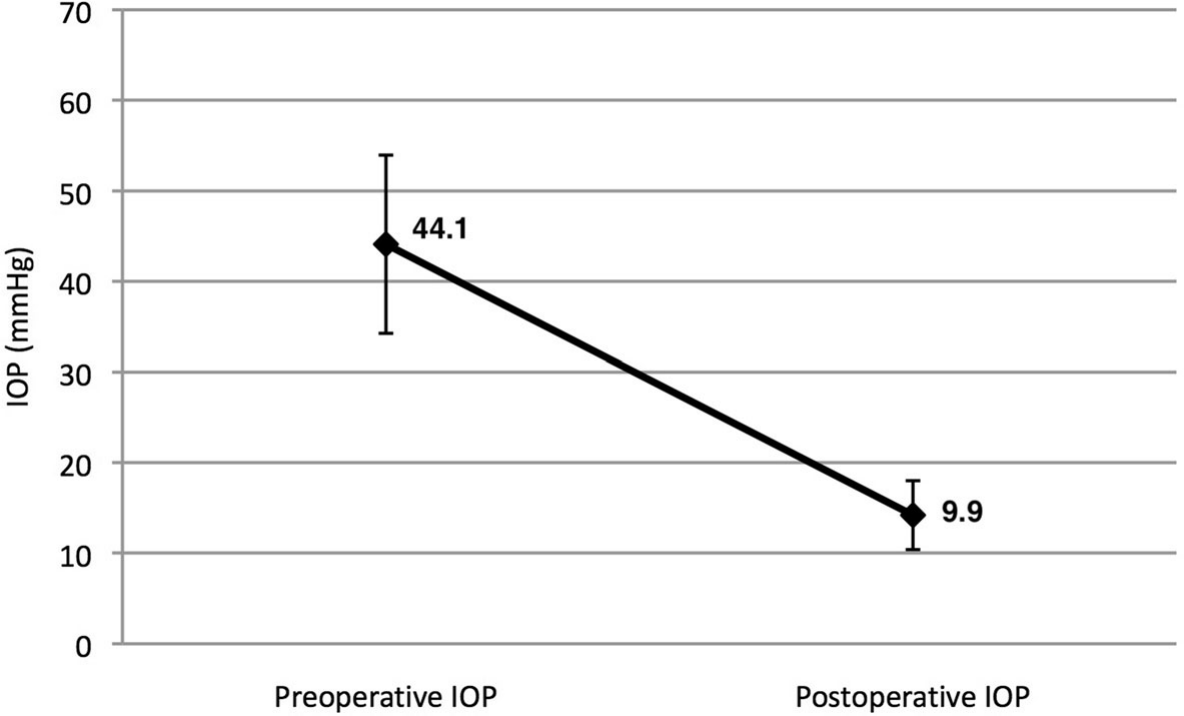
The mean preoperative and postoperative intraocular pressure (IOP) of 27 study patients. Error bars indicated standard deviations

The mean IOP, which was 44.1 mmHg (range 26–62) before the operation, decreased to 14.2 (range 8–20) mmHg at the last follow-up visit, showing 67% reduction with AGV implantation (Fig. 2). The mean postoperative IOP during the follow-up period was 22.6 mmHg (range 10–35) (Table 2). Eleven (40.7%) cases with IOP over 18 mmHg required additional treatment: 1 required diode cyclophotocoagulation and 10 were on 1–4 glaucoma medications (Table 1, Table 2).

**Fig. 3:**
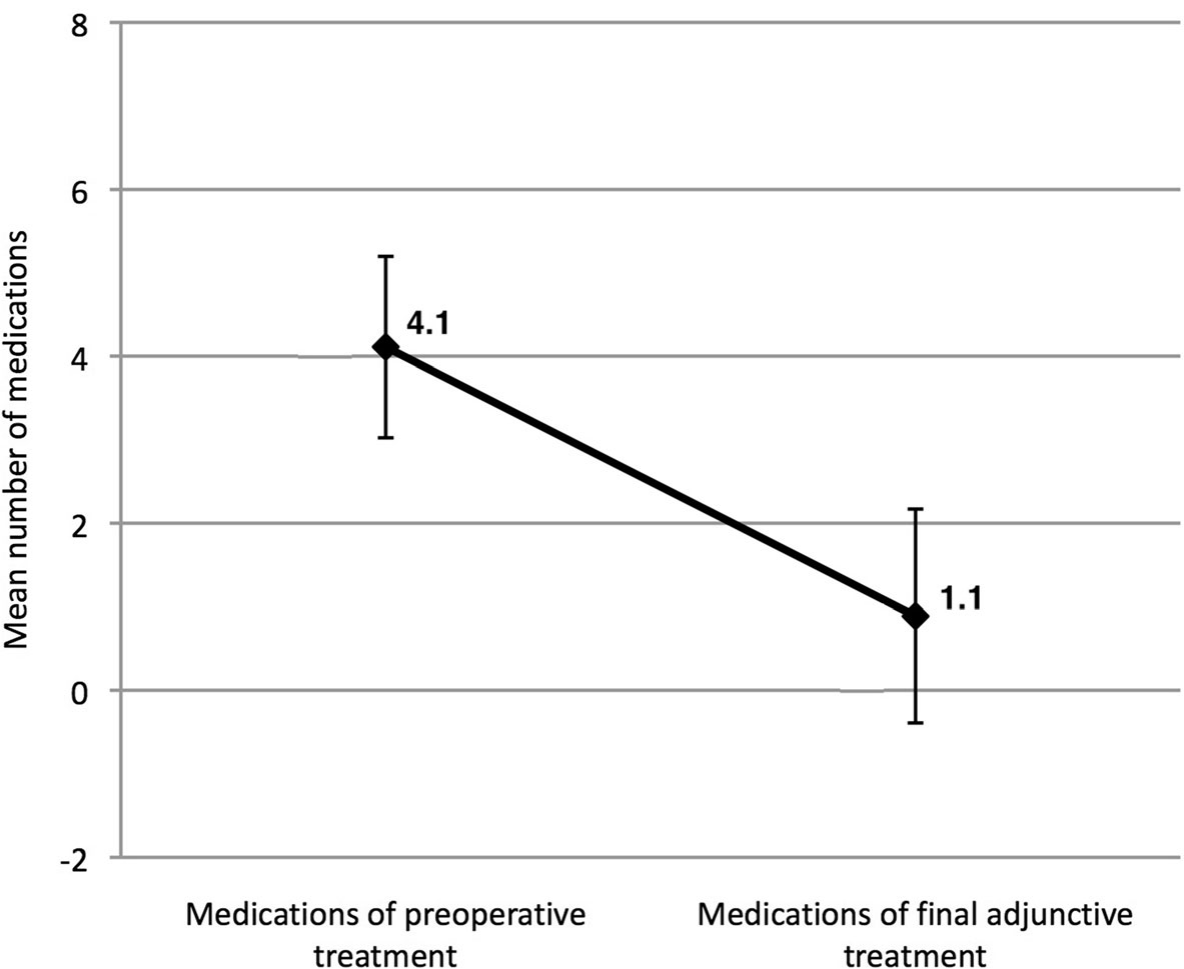
The mean number of preoperative and postoperative medications used for 27 eyes. Error bars indicated standard deviations

The mean number of anti-glaucomatous medications, which was 4.1 before the AGV implantation, decreased to 0.9 after the operation, showing 88% reduction (Table 2, Fig. 3].

As a complication of AGV implantation, postoperative hyphema developed in 2 patients (7.4%), requiring AC lavage. In 3 eyes, the tube was occluded by the iris needed for tube repositioning, uveitic inflammatory debris, and silicon oil. In 2 eyes with neovascular glaucoma and proliferative diabetic retinopathy, intravitreal hemorrhage developed postoperatively and additional intravitreal anti-VEGF and pars plana vitrectomy were required. In 1 eye, choroidal detachment resolved spontaneously. In 1 eye, suprachoroidal hemorrhage and choroidal detachment necessitated pars plana vitrectomy and silicone oil injection. Tenon’s cysts developed in 2 eyes that resisted drainage and required cystectomy. In 2 eyes, secondary cataract formation required phacoemulsification (Table 1).

No patient developed postoperative hypotony, flat anterior chamber, diplopia, strabismus, erosion or exposure of the tube, or tube/plate migration.

## Discussion

In the present study, we described a novel technique of creating a long scleral flap augmented with Tenon advancement and duplication to prevent exposure of the AGV tube in patients with refractory glaucoma. Our experience with 27 eyes indicated that this technique is effective in decreasing the IOP and requirement for antiglaucomatous medications without adversely affecting BCVA and not causing erosion or exposure of the tube or tube/plate migration. Therefore, we suggest that the long scleral flap augmented with Tenon advancement and duplication is an effective and safe surgical technique for the implantation of AGV and prevention of tube exposure in cases with refractory glaucoma.

The AGV was developed in 1993 and has become the most commonly used glaucoma drainage device with high success rates of 60–90% in 1 year and 40–50% in up to 4 years [1, 10–14]. It has been particularly used for the management of glaucoma refractory to standard filtering surgery. Despite its well-documented efficacy in the treatment of refractory glaucoma, various early and late postoperative complications have been reported with AGV implantation, one of which is exposure of the tube through conjunctival erosion. Since the exposed tube gives easy access to microorganisms into the eye, it may lead to serious complications such as ocular inflammation, hypotony, poor vision, phthisis, and endophthalmitis [2–6]. Tube exposure is usually the result of continuous microtrauma to the conjunctiva, which induces inflammatory and/or immunologically mediated tissue damage. Younger age, previous inflammation, diabetes, and inferiorly placed implants are well-known risk factors for tube exposure associated with glaucoma drainage devices [7, 15, 16]. AGV, Baerveldt, and Molteno implants do not show significant differences in terms of tube exposure rates [17].

Although various surgical techniques have been described to prevent tube exposure, such as placement of patch graft (e.g., fascia lata, pericardium, donor sclera, or lyophilized dura mater patch grafts), long scleral tunnel, and/or doubling and advancement of Tenon’s tissue [8, 18–22], there is no consensus on the ideal technique. Our technique is AGV implantation using a long scleral flap augmented with Tenon advancement and duplication. The rationale behind this technique is to form two natural barriers, which are the sclera and Tenon’s tissues, which keep the silicon tube away from the conjunctiva throughout the whole tube tract, providing protection from mechanical trauma. The other advantage of this method is that it prevents inflammation or immunological reaction against foreign materials such as grafts. Besides, the placement of the tube under the long scleral flap helps prevent mechanical insult of the tube bending at the proximal 3-mm corneoscleral limbus, providing a smooth tract for the tube. Fixing the tube under the sclera also prevents microdamage due to continuous movement of the tube.

In the patch graft technique, although tube exposure is significantly reduced, there is still a 5–14% risk of tube exposure regardless of the graft and glaucoma drainage device type used [15, 16]. However, with our technique, the exposure rate was 0% during an average of 21.7 months of follow-up. Furthermore, considering the disadvantages of various patch grafts like the risk of melting, mechanical trauma, immune atrophy and late exposure, infection, and rejection, along with the high cost of fibrin glue fixation [7, 15, 23, 24], our technique can be considered superior to patch graft technique. In our technique, as we did not use any foreign material, there was no risk of inducing an immunologic reaction. As the scleral flap was sutured over the tube, there was no continuous micromovement of the conjunctiva over the tube and no risk of mechanical trauma that leads to erosion. Our sutures in the scleral flap were under the duplicated Tenon’s tissue, and did not carry the risk of infection owing to their long distance from the ocular surface. The use of autologous material has the advantages of absence of immunologic reactions and low cost.

In a meta-analysis by Stewart et al. [17], previously published studies describing conjunctival erosion in patients with a glaucoma device (16 AGVs, 12 Baerveldt, and 17 Molteno implants) showed that tube exposure is not a very late complication of the glaucoma drainage device surgery. A total of 3105 patients and 3255 eyes with an average follow-up of 26.1 ± 3.3 months were included in the analysis with the incidence of tube exposure 2.0% ± 2.6% (*n* = 64), and an average exposure rate per month of 0.09% ± 0.14%. Although the correlation between study length and incidence of exposure was not significant, there appeared to be a little increase in exposure incidence for studies up to 2-year follow-up [17]. Based on this meta-analysis, we conclude that our mean follow-up duration is sufficient enough to evaluate the effect of this technique on the development of tube exposure.

Similar to the findings of previous studies [7, 15, 16], our study population had no risk factors for tube exposure such as younger age, inflammation prior to tube exposure, and diabetes. Since we had no case with an inferiorly placed AGV implant, we could not determine the tube exposure rate of this novel technique in patients with inferiorly placed implants. We can only report that there was no difference between the superotemporal quadrant and the superonasal quadrant placement in our study.

Another previous technique of preventing tube exposure involves the use of a long scleral tunnel for the implantation of anterior tube parts of the glaucoma drainage device [18, 19]. Long scleral tunnel technique was found to be superior to the patch graft method in preventing tube exposure after AGV implantation [25]. However, the long scleral tunnel technique has the limitation of a long tunnel which complicates advancing the lancet through the curved contour of the sclera. Moreover, the entrance to the anterior chamber with a lancet is more traumatic and carries higher risk of leakage around the tube than entrance with a 23-gauge needle. In our novel technique, we created a half-thickness smooth flap in the sclera, so direct visualization of the length of the scleral bed provided a more delicate tract and alleviated the risks posed by the blinded curved tunnel technique. We entered the anterior chamber using a 23-gauge needle safely with direct visualization.

Some previous studies reported successful results with the short scleral tunnel technique even in cases of congenital glaucoma [26]. However, we did not prefer the short scleral tunnel or flap because we think that covering the entire length of the tube is the nontraumatic route and burying the tube completely in the sclera prevents the bending of the proximal tube along the curvature of the globe.

Brouzas [27] presented the double scleral tunnel in tandem technique, offering two short tunnels in order to overcome the length limitations of one tunnel and completely burying the tube in the sclera, but tube exposure through conjunctival erosion still occurred in 7.1% of the cases in this study.

Tamcelik [28] described a technique that combined short scleral tunnel with Tenon advancement and duplication and reported no tube exposure even at follow-up of 8 years. Our technique has the practical advantages of Tamcelik’s technique and provides even lower complication rates. In our technique, we made a watertight suture on the flap along the path of the tube, so there was a lower risk of leakage and subsequent hypotony. None of our patients experienced postoperative hypotony or shallow anterior chamber. A large proportion of our patients experienced a significant improvement in their visual acuity. In the vast majority of our patients, visual acuity was low due to advanced glaucomatous optic neuropathy. The relative increase in postoperative BCVA was the result of the resolution of the preoperative corneal edema along with a decrease in intraocular pressure.

The possible limitation of our technique is the size of the filtration lake formed around the plate due to the pulling up and shortening of the Tenon’s capsule. Thinning of the Tenon layer by aging may cause failure of the surgery making the technique inapplicable. This should be clarified with further long-term and large studies. Our technique may not be ideal for all cases as scleral tissue may be very thin in patients who have previously undergone multiple surgeries.

## Conclusions

In conclusion, the proposed long scleral flap augmented with Tenon advancement and duplication is an effective and safe surgical method for the implantation of AGV and preventing tube exposure in patients with refractory glaucoma. It is also an easy technique with low cost. Although the rate of complications associated with this technique is low, we suggest that this new surgical technique be further explored by surgeons in order to determine its long-term safety and efficacy.

## Abbreviations

AC: Anterior chamber
AGV: Ahmed glaucoma valve
BCVA: Best corrected visual acuity
IOP: Intraocular pressure
